# Supplementary material for: Synthesis and Properties of Plasma-Polymerized Methyl Methacrylate via the Atmospheric Pressure Plasma Polymerization Technique
Source: Polymers (Basel). 2019 Feb 28;11(3):396. doi: 10.3390/polym11030396 (PMC6473653; doi:10.3390/polym11030396)
Supplement: Supplementary file 1 [file polymers-11-00396-s001.pdf]

polymers

MDPI

Supporting Information

Synthesis and Properties of Plasma-Polymerized Methyl Methacrylate via Atmospheric Pressure Plasma Polymerization Technique

Choon-Sang Park [1], Eun Young Jung [1], Hyo Jun Jang [1], Gyu Tae Bae [1], Bhum Jae Shin [2] and Heung-Sik Tae [1,*]

[1] School of Electronics Engineering, College of IT Engineering, Kyungpook National University, Daegu 41566, Korea; purplepcs@ee.knu.ac.kr (C.-S.P.); eyjung@knu.ac.kr (E.Y.J.); bs00201@knu.ac.kr (H.J.J.); doctor047@knu.ac.kr (G.T.B.)

[2] Department of Electronics Engineering, Sejong University, Seoul 05006, Korea; hahusbj@sejong.ac.kr

* Correspondence: hstae@ee.knu.ac.kr; Tel.: +82-53-950-6563

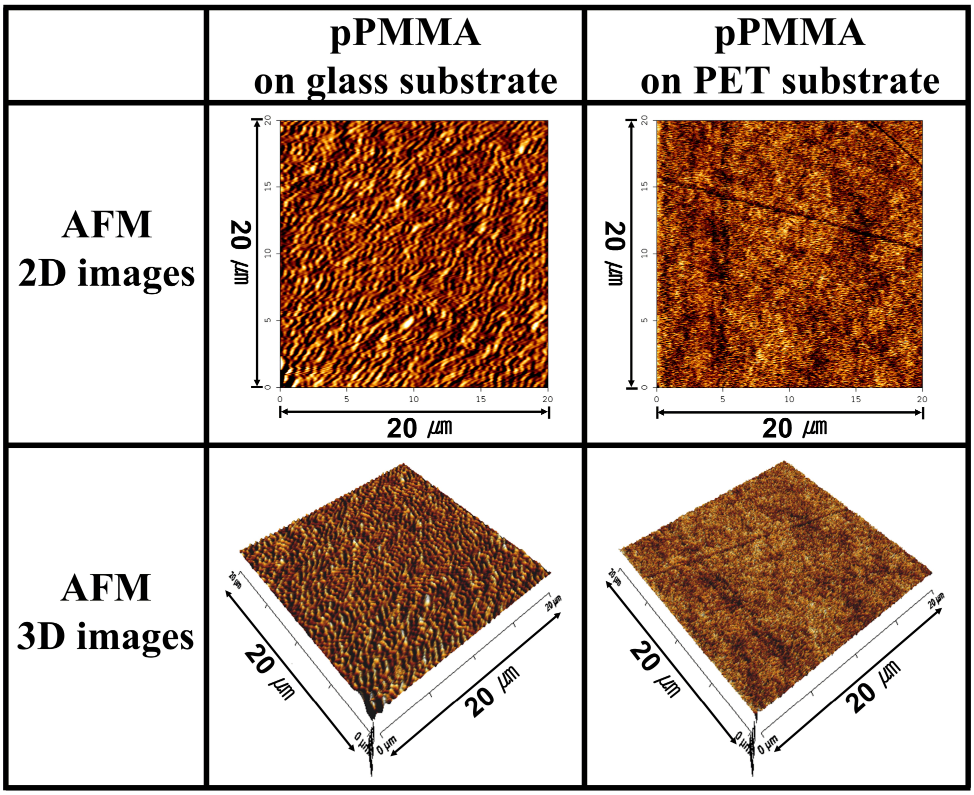

Figure S1. Two- (2D) and three-dimensional (3D) AFM images of pPMMA film surfaces grown on glass and PET substrates when using proposed APP polymerization technique after 90 min deposition.

Figure S1 and Table S1 show the changes in two- (2D) and three-dimensional (3D) AFM images according to the root mean square roughness (R_q) and average roughness (R_a) of pPMMA film surfaces grown on glass and PET substrates when using proposed APP polymerization technique after 90 min deposition. The surface roughness of the pPMMA films was performed on a non-contact mode by Atomic Force Microscopy (Brucker, NanoWizard II, Germany) at the Korea Basic Science Institute (KBSI; Busan). All measurements were obtained under controlled room temperature. Moreover, the scanning area was 20 μm × 20 μm and scan rate was set at 1 Hz. The Bruker

NanoWizard software was used for image processing and interpretation. The surface roughness (root mean square roughness, R_q) of the pPMMA thin films on the glass substrate was 25.9 nm, whereas the R_q of the pPMMA thin films on the PET substrate was 0.6 nm in Figure S1 and Table S1. The roughness of the pPMMA thin films on both glass and PET substrates was changed; this changed roughness after 90 min deposition was mainly due to differences of surface energy and Young's modulus between various pristine substrates.

Table S1. Root mean square roughness (R_q) and average roughness (R_a) obtained from AFM images of pPMMA film surfaces grown on glass and PET substrates when using proposed APP polymerization technique after 90 min deposition.

**Sample**	**pPMMA on glass**	**pPMMA on PET**
R_q	25.9 nm	0.6 nm
R_a	19.9 nm	0.4 nm

© 2019 by the authors. Submitted for possible open access publication under the terms and conditions of the Creative Commons Attribution (CC BY) license (http://creativecommons.org/licenses/by/4.0/).
